# Beyond Competencies: Associations between Personality and School Grades Are Largely Independent of Subject-Specific and General Cognitive Competencies

**DOI:** 10.3390/jintelligence10020026

**Published:** 2022-04-27

**Authors:** Lena Roemer, Clemens M. Lechner, Beatrice Rammstedt

**Affiliations:** GESIS—Leibniz Institute for the Social Sciences, P.O. Box 12 21 55, D-68072 Mannheim, Germany; clemens.lechner@gesis.org (C.M.L.); beatrice.rammstedt@gesis.org (B.R.)

**Keywords:** Big Five, personality, academic achievement, school grades, cognitive ability, competence

## Abstract

The Big Five personality traits are established predictors of school grades. However, the mechanisms underlying these associations are not yet well understood. Effects of personality on grades might arise because behavioral tendencies facilitate learning and increase subject-specific competencies. Alternatively, personality effects on grades might be independent of cognitive competencies and reflect otherwise valued behaviors or teachers’ grading practices. In the current study, we drew on large-scale data of 7th and 9th graders in Germany to explore the extent to which personality predicted grades even after accounting for competencies. Controlling for competencies and other key covariates, we cross-sectionally and longitudinally examined personality–grade associations across different school subjects, grade levels, and school types. Results indicate that the predictive power of personality is largely independent of subject-specific and general cognitive competencies. The largest effects emerged for conscientiousness. For openness, associations with grades partly overlapped with competencies, suggesting that openness may operate by fostering competencies. Overall, our results suggest that the associations between personality and grades unfold mostly independently of course mastery. This finding underlines the socioemotional value of personality in the classroom and encourages a more fine-grained view of the interplay between personality, competencies, classroom behavior, and grades.

## 1. Introduction 

Personality is robustly associated with academic achievement (Borghans et al. 2016; Lavin 1965; Lechner et al. 2017; Mammadov 2021; Poropat 2009; Spengler et al. 2013; Zhang and Ziegler 2016). Conscientiousness in particular predicts school grades and scores on standardized achievement tests (e.g., Noftle and Robins 2007). However, the psychological mechanisms underlying these personality–achievement associations still need to be better understood. In particular, the associations between personality and school grades are assumed to reflect multiple mechanisms (Hübner et al. 2022; Poropat 2009; Westphal et al. 2021). On the one hand, specific behavioral tendencies might facilitate learning, fostering the competencies a student needs, for example, in mathematics and thus lead to better school grades. On the other hand, personality–grade associations might also reflect behavioral tendencies that are not required for competence mastery but that are still reflected in teachers’ grading practices (e.g., Westphal et al. 2021). 

In the current study, we examine the associations between personality and school grades. Using large-scale data from *N* = 7861 7th and 9th graders in Germany, we aim to disentangle the extent to which personality–grade associations reflect or are independent of subject-specific cognitive competencies. We explore whether personality predicts grades even after controlling for subject-specific and general cognitive competencies and other covariates, and we do so across different subjects, grade levels, temporal perspectives, and school types. Triangulating these results, we seek to shed light on the criterion-related validity of personality for predicting grades.

### 1.1. Academic Achievement and Personality 

Individual differences in thinking, feeling, and behaving, as described by the Big Five personality traits (Goldberg 1993), can predict differences in academic success. Such associations between personality and academic achievement exist even beyond the influence of general cognitive ability (e.g., Barbaranelli et al. 2003; Borghans et al. 2016; Lechner et al. 2017; Mammadov 2021; Meyer et al. 2019; Poropat 2009; Spengler et al. 2013; Zhang and Ziegler 2016; Ziegler et al. 2012), the central predictor of academic achievement (Deary et al. 2007). In particular conscientiousness and—to a lesser extent—openness have been established as robust predictors of academic achievement (e.g., De Raad and Schouwenburg 1996; Dumfart and Neubauer 2016; Hübner et al. 2022; Mammadov 2021; Poropat 2009). Conscientiousness represents a person’s self-discipline and will to achieve and their tendency to responsibly keep things in order and follow rules (e.g., Soto and John 2017). Openness represents a person’s tendency to seek out and engage with new input, fostering creativity and intellectual curiosity. A recent meta-analysis estimated the average relations of conscientiousness and openness with academic achievement to be *ρ* = .27 and .16, respectively (Mammadov 2021). For the remaining Big Five traits, the relations to academic achievement are weaker or still inconclusive (.01 ≤ |*ρ*| ≤ .09; Mammadov 2021). 

#### 1.1.1. Personality Is Differentially Associated with Academic Achievement

The associations between personality and achievement differ across achievement measures (Borghans et al. 2016; Hübner et al. 2022; Lechner et al. 2017). Academic achievement is an umbrella term, with different measures representing substantially different aspects. Two types of measures are commonly distinguished: standardized achievement tests and teacher-assigned grades, which tend to be only moderately related (e.g., Borghans et al. 2016; Willingham et al. 2002). Standardized achievement tests aim to objectively assess domain-specific competencies and knowledge relevant for succeeding in academic or daily life (e.g., OECD 2009). Owing to their standardized nature, achievement tests are considered a relatively pure measure of domain-specific cognitive competencies (cf. Hübner et al. 2022). By contrast, teacher-assigned grades typically reflect a broader set of influences beyond subject-specific competencies. Most importantly, they contain information about teachers’ subjective evaluations of student performance, classroom behavior, and motivation (Spengler et al. 2013; Willingham et al. 2002). 

Generally, personality is more closely related to grades than to standardized achievement scores. For example, the Big Five explained up to 18% of the variance in grades in 9th graders in Germany (.05 ≤ *R*^2^ ≤ .18) and substantially less in standardized achievement scores (.03 ≤ R^2^ ≤ .06; Brandt et al. 2020; see also Borghans et al. 2016; Israel et al. 2019; Lechner et al. 2017). 

#### 1.1.2. Associations between Personality and Academic Achievement Might Reflect Different Mechanisms 

The differential predictive power of personality across measures of academic achievement suggests that personality–achievement associations reflect different psychological mechanisms, whose importance may differ across achievement measures. The mechanisms that underlie personality–achievement associations can broadly be described as representing two paths: a competence-related path and a competence-independent path.

Personality–achievement associations are commonly assumed to reflect the competence-related path. Specific traits and behaviors—asking questions in case of a lack of understanding, repeating vocabulary regularly, thinking outside the box—are assumed to foster learning and academic mastery (e.g., Cupani and Pautassi 2013; De Raad and Schouwenburg 1996; von Stumm 2018; Ziegler et al. 2018). Exemplifying the competence-facilitating potential of personality, the Big Five-Narrow Traits Model (Zhang and Ziegler 2016) proposes that the effects of personality on grades operate via specific learning approaches and subject-specific academic self-beliefs. Supporting this assumption, Zhang and Ziegler (2016) found, for example, that Chinese students high in openness had positive subject-specific academic self-concepts, used deep learning strategies, and thus received better grades in mathematics and Chinese. The competence-facilitating potential of personality is also recognized in the personality–achievement saturation hypothesis (PASH; Hübner et al. 2022). PASH posits that different features of achievement measures require different personality traits. For less instructionally sensitive measures, such as standardized achievement tests, openness is assumed to be particularly predictive. Open behaviors, such as seeking out new problems and applying learning strategies, can improve students’ problem-solving abilities and enable them to score high on competence tests (see also Ziegler et al. 2012). 

However, personality–achievement associations might also operate independently of competencies and cognitive abilities (Poropat 2014b; Spengler et al. 2013; Westphal et al. 2021). Such competence-independent effects have been posited to reflect the social value of behaviors, halo effects in grading, or implicit assumptions about what “a good student” should be like (Poropat 2014a; Spengler et al. 2013). For instance, conscientious and open classroom behavior, such as careful note taking and curious questioning, is viewed as beneficial from a teacher’s perspective (e.g., Barbaranelli et al. 2003; Poropat 2014b). These behavioral tendencies might thus also relate to academic achievement because of the value that they have in the eyes of the teacher. Exemplifying this competence-independent path, Zhang and Ziegler (2016) found incremental effects of personality on grades, even beyond motivational and cognitive mediators. Similarly, as postulated in PASH (Hübner et al. 2022), the relevance of conscientiousness for instructionally sensitive measures such as teacher-assigned grades could result from conscientious classroom behavior being appreciated by teachers. 

Overall, while the competence-related path makes personality a potentially promising target for educational interventions, the competence-independent path highlights that associations between personality and academic achievement might also reflect mechanisms other than cognitive competencies. 

#### 1.1.3. Associations between Personality and School Grades Might Reflect Different Mechanisms

Particularly for school grades, the associations with personality and academic achievement have been argued to reflect a conglomerate of the competence-related and competence-independent path (e.g., Spengler et al. 2013; Steinmayr et al. 2011). In fact, this was recently shown for 9th graders in Germany: In a cross-sectional study, Westphal et al. (2021) explored whether personality–grade associations operated via subject-specific competencies or existed independently of competencies. For openness, the associations with grades were (partially) mediated by subject-specific competencies, arguably reflecting competence-related contributions of personality. Remarkably, however, the lion’s share of the predictive power of personality for grades was independent of subject-specific competencies. This suggests that the associations between personality and grades are mediated only to a limited extent by learning behavior or competence gains. Instead, personality–grade associations seem to primarily reflect factors other than competencies, such as teachers’ grading practices, halo effects, or the social value of certain traits. 

Empirically distinguishing the competence-related and competence-independent contributions of personality thus puts to the test a central assumption about the mechanisms underlying the links between personality and grades (Westphal et al. 2021). We next outline why a rigorous exploration of these contributions should include additional aspects such as different temporal perspectives or the contributions’ generality across academic domains. 

### 1.2. Rigorously Exploring the Competence-Related and Competence-Independent Associations between Personality and Grades 

#### 1.2.1. Temporal Perspective: Do the Competence-Related and Competence-Independent Associations Exist Cross-Sectionally and Longitudinally?

The finding that personality makes large competence-independent contributions to grades comes from cross-sectional data (Westphal et al. 2021). The large competence-independent contributions could thus be argued to be overestimated—inflated by the concurrent assessment of personality and grades and a possibly short-lived impact of grading practices. Personality–grade associations have only rarely been studied longitudinally. Such studies have indicated relatively small effects of personality on later grades (.01 ≤ |β| ≤ .11; Israel et al. 2019; see also Spengler et al. 2016). However, how the competence-related and competence-independent contributions of personality may unfold over time remains unexplored. Longitudinal data could inform as to whether longitudinal relations between personality and grades reflect change in competencies or rather more persistent grading practices.

#### 1.2.2. Grade-Level Differences: Do the Competence-Related and Competence-Independent Associations Exist across Different Grade Levels?

Relations between personality and grades can differ across grade levels (e.g., Andersen et al. 2020; Laidra et al. 2007; Poropat 2009). For example, comparing samples of 6th and 9th graders, Tetzner et al. (2020) found that personality–grade links were weaker among 9th graders than among 6th graders for extraversion (*d* = −.22), stronger for neuroticism (*d* = .13), and similar for the remaining dimensions. This also suggests that the competence-related and competence-independent contributions of personality to grades may differ across grade levels. Considering such grade-level differences could shed light on potential developmental demands reflected in personality–grade associations. 

#### 1.2.3. Generalizability: Do the Competence-Related and Competence-Independent Associations Exist across School Subjects?

Consistent with other research (e.g., Brandt et al. 2020; Meyer et al. 2019), the competence-independent and competence-related contributions of personality were partly subject-specific (Westphal et al. 2021). For example, the competence-independent contribution of openness was positive for grades in German but negative for grades in mathematics, suggesting the same behavioral tendencies to be differentially valued across subjects. Testing the competence-related and competence-independent associations of personality with grades across academic domains informs about the subject-specificity of these associations. In addition to languages and mathematics, science subjects are another key academic domain (OECD 2009). Yet, less is known regarding the associations between personality and science subjects (cf., e.g., Dumfart and Neubauer 2016). A study taking into account the three academic domains could thus help to explore the generality of competence-related and competence-independent contributions of personality. 

#### 1.2.4. Specificity: Do the Competence-Related and Competence-Independent Associations Exist beyond Covariates?

General cognitive abilities are related to both academic achievement (Deary et al. 2007) and personality (Ackerman and Heggestad 1997), making them a possible confounder of personality–grade associations. Neglecting to control for general competencies could inflate the estimation of competence-independent associations of personality with grades. Additionally, key sociodemographic covariates—sex, migration background, and socioeconomic status (Damian et al. 2015; Feingold 1994; Lechner et al. 2021)—should also be controlled for as potential confounders (e.g., as in Westphal et al. 2021). 

### 1.3. The Current Study

In the current study, we explored the predictive power of personality for grades. Do personality–grade associations reflect behavioral tendencies that might facilitate learning and result in competence mastery? Or are they independent of competencies, suggesting that they reflect otherwise valued behavioral tendencies or potentially teachers’ more subjective grading practices? 

We examined the competence-related and competence-independent contributions of personality to grades using large-scale data from German students (Starting Cohort 3 of the German National Education Panel Study, NEPS; Blossfeld et al. 2011), triangulating various analyses. First, we adopted a cross-sectional and longitudinal perspective to explore both the concurrent and longitudinal interplay between personality, competencies, and grades. Second, to take differences across grade levels into account, we examined the competence-related and competence-independent contributions for the same sample in 7th and 9th grade. Third, to assess generality across academic domains, we analyzed the relations to grades in German, mathematics, and science. Fourth, we controlled for general cognitive competencies and sociodemographic covariates as potential confounders. Finally, we tested the robustness of the results and explored the contributions (a) across school types, as school type has been shown to moderate the association between personality and achievement (Brandt et al. 2020); (b) accounting for referential grading practices (“grading on a curve”, e.g., Calsamiglia and Loviglio 2019); and (c) accounting for unreliability (Westfall and Yarkoni 2016).

We examined the contributions of personality to grades for all Big Five domains. For conscientiousness and openness, we preregistered positive effects on grades to remain when controlling for competencies—and thus to also reflect the competence-independent path. For the remaining domains, we did not preregister any hypotheses, but rather studied the competence-related and competence-independent contributions in a more exploratory way.

## 2. Method

### 2.1. Participants

**Figure 1:**
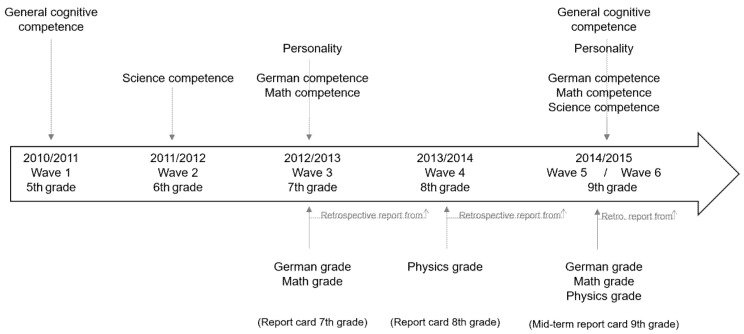
Overview of the timing of the assessment of the constructs.

We used data from the German National Education Panel Study (NEPS; Blossfeld et al. 2011). NEPS is a large-scale multicohort, multiwave study on competence development over the life course. We focused on Starting Cohort 3, which is a representative sample of students in Germany who were followed from 5th grade onward and first assessed in 2010. Apart from excluding students attending special schools, we applied no exclusion criteria. We used data from Wave 1 to Wave 6 (see Figure 1). We included all participants who provided data on at least one of the variables or covariates under study in at least one of the waves covered, leading to a final sample of *N* = 7861 participants (48% female)^1^. In 7th grade (Wave 3, the first wave with a personality assessment), the average age of the participants was *M* = 12.93 years (*SD* = 0.53); 49% were in an academic school track (*Gymnasium* or *Gymnasialzweig*). 

### 2.2. Measures

Descriptive statistics and bivariate correlations among all measures are displayed in Supplementary Material Table S1. 

#### 2.2.1. Personality 

Personality was assessed using a short version of the Big Five Inventory, the BFI-10, (Rammstedt and John 2007), which is widely used in large-scale studies. The measure was administered in 7th and 9th grade. The BFI-10 measures each domain with a positively and negatively coded item, allowing to control for acquiescence at the scale score level. The two items per domain were selected largely to capture the construct breadth. A third agreeableness item was added to improve the breadth and reliability of that scale (see Rammstedt and John 2007). Items had a 5-point rating scale (1 = *does not apply at all*, 5 = *applies fully*). McDonald’s omega ranged between .42 (agreeableness) and .58 (conscientiousness) in 7th grade and between .39 (agreeableness) and .64 (extraversion) in 9th grade. Varimax rotated principal component analyses (see, e.g., Soto and John 2017) supported the five-factor structure of the scale scores in both 7th and 9th grade. The items loaded highest on the respective domain factor. A single exception occurred for the negatively coded item for agreeableness, which, in both 7th and 9th grade, showed higher (cross-)loadings on other domain factors, particularly on conscientiousness. For each domain, we calculated unit-weighted mean scores. We opted for this scoring approach to represent the constructs as broadly as possible. With an alternative latent variable approach, the common variance of each item pair would have been extracted, which would have run counter to the scale’s construction rationale to select distinct items to represent the breadth of each domain.

#### 2.2.2. Cognitive Competencies

**Subject-Specific Competencies.** Subject-specific competencies were assessed using standardized achievement tests. In NEPS, these tests combine the competence framework of the PISA studies (OECD 2009) with German national educational standards (e.g., Hahn et al. 2013; Neumann et al. 2013). Competence test items are scaled within an item response theory framework. Scale scores are provided as weighted likelihood estimates (WLE). Competence tests in NEPS are age-tailored, but scores can be linked over time. To allow for comparisons over time, we used WLE scores linked to underlying reference scales (Fischer et al. 2016).

Linguistic competence in German was assessed through a reading comprehension test (Gehrer et al. 2013) and a spelling test (Blatt and Prosch 2014), administered both in 7th and 9th grade. The reading test focuses on the ability to read different types of text (e.g., instructional texts) and to use text appropriately (e.g., find information; Gehrer et al. 2013). WLE reliability was .79 in both 7th and 9th grade (Krannich et al. 2017; Scharl et al. 2017). The spelling test assesses orthography using a cloze test and full sentences. WLE reliability was .94 in both 7th and 9th grade (Blatt et al. 2017). As an overall German linguistic competence score, we calculated a mean score of the standardized WLE scores for reading and spelling.

To assess mathematical competence, NEPS uses a test focusing on the ability to apply cognitive processes (e.g., mathematical argumentation) across content areas (e.g., data and chance) to solve mathematics-related problems (Neumann et al. 2013). The test was administered in 7th and 9th grade. The WLE reliability of the mathematics test scores was .72 in 7th grade and .81 in 9th grade (Schnittjer and Gerken 2017; van de Ham et al. 2018).

Scientific competence was assessed with a scientific literacy test (Hahn et al. 2013). This test focuses on the ability to apply content-related scientific knowledge (about physics, chemistry, and biology) and process-related knowledge (e.g., about scientific reasoning) to everyday life problems. Scientific literacy was assessed in 6th and 9th grade. The WLE reliability of the test scores was .77 and .80, respectively (Funke et al. 2016; Kähler 2020).

**General Cognitive Competencies.** We used the sum score of the NEPS matrices test (Haberkorn and Pohl 2013) to assess reasoning ability as an indicator of general cognitive competencies. Based on Raven’s Matrices, this test assesses the ability to complete figural matrices by deduction. It consists of 12 matrices for which students must select the element that completes the underlying pattern. The test was administered in 5th and 9th grade. Kuder Richardson internal consistency estimates were KR20 = .66 in 5th grade and .65 in 9th grade. 

#### 2.2.3. School Grades

The central outcome was teacher-assigned grades as reported by the students for German, mathematics, and science/physics.^2^ In the German school system, grades range from 1 (*very good*) to 6 (*insufficient*). We recoded grades such that higher values meant better achievement. For each subject, we selected the grades that most closely followed the respective competence assessment (see Figure 1). For German and mathematics (competence tests in 7th and 9th grade), we used grades from the final report card for 7th grade and from the mid-term report card for 9th grade. For science (competence tests in 6th and 9th grade), we used physics grades from the final report card for 8th grade and from the mid-term report card for 9th grade. 

#### 2.2.4. Covariates

As sociodemographic covariates, we considered sex (0 = male, 1 = female); migration background (0 = no migration background, 1 = born abroad or both parents born abroad); and (highest) parental socioeconomic status (International Socio-Economic Index of Occupational Status (ISEI-08) scores ranging between 10 and 90; scores were based on parental reports of their jobs and reflect information on the average job holder’s income and education). Based on the findings by Brandt et al. (2020), we also considered school type (0 = academic track; 1 = non-academic track) as a potential moderator of the personality–grade associations.

### 2.3. Data Analysis 

We preregistered the analysis plan on the Open Science Framework (OSF) prior to the analyses.^3^ To assess the concurrent relations between personality and grades, we analyzed hierarchical multiple regressions with school grades as the outcome variables. We built the models in a stepwise manner, representing an increasingly rigorous test of the relation between personality and grades. First, we included all scale scores for the Big Five domains (models M1); second, we controlled for subject-specific and general cognitive competence scores (M2); and third, we included sociodemographic covariates (M3). We analyzed separate models for German, mathematics, and science, and for 7th and 9th grade (i.e., a total of 18 models: 3 stepwise models × 3 subjects × 2 grade levels).

To assess the longitudinal relations between personality and grades, we estimated first-difference models (e.g., Liker et al. 1985). The structure of these models resembled that of the cross-sectional models. However, criteria and predictors were entered as difference scores across the two timepoints (e.g., the German grade in 7th grade was subtracted from the German grade in 9th grade). First, we predicted change in grades from change in personality scores separately for German, mathematics, and science (M1_longi_). Second, we additionally controlled for change in subject-specific and general cognitive competence scores (M2_longi_). With their focus on change, first-difference models implicitly control for the influence of (unobserved) time-invariant covariates (e.g., Liker et al. 1985). We therefore did not include the (time-invariant) sociodemographic covariates in these models. 

We specified all models within a path analysis framework. We adapted three measures to mitigate potential bias due to selective dropout, thereby deviating from the preregistered analysis plan. First, to account for missing data, we used (robust) full information maximum likelihood estimation (FIML). Second, to ensure sample comparability across the three stepwise cross-sectional models, we included the sociodemographic covariates from model M3 as auxiliary variables in models M1 and M2 (Enders 2008). Third, to also include in the longitudinal analyses students who provided data on only one measurement occasion, we estimated the difference scores as pseudo-latent difference variables within the path models.^4^

## 3. Results 

### 3.1. Cross-Sectional Analyses 

**Figure 2:**
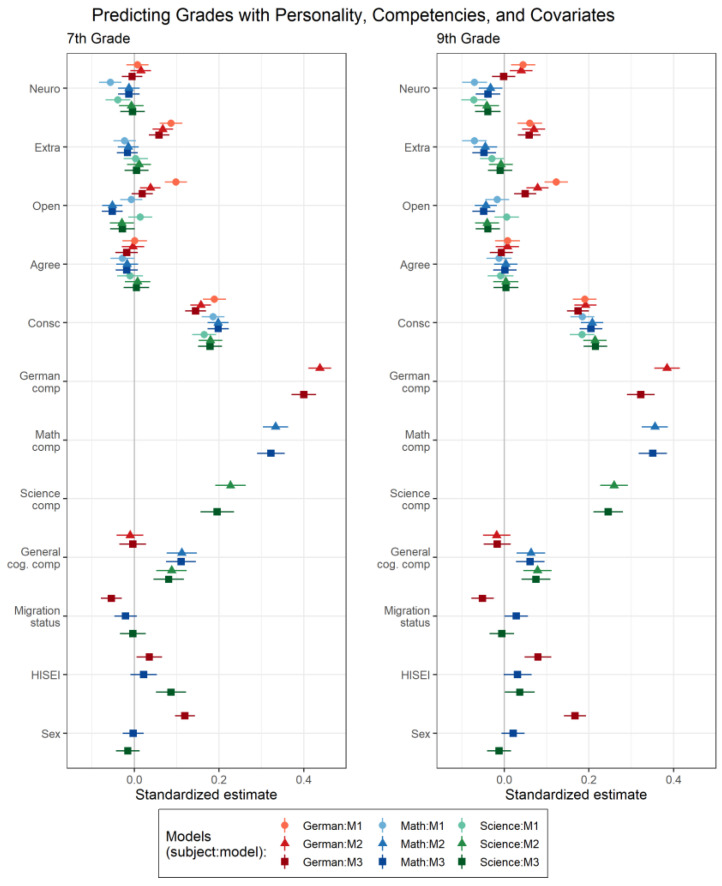
Standardized estimates for the cross-sectional models. Results are shown for the increasingly comprehensive models M1–M3 separately for the subjects and grade levels. Neuro = neuroticism; Extra = extraversion; Open = openness; Agree = agreeableness; Consc = conscientiousness; comp = competence; cog = cognitive; HISEI = Highest International Socio-Economic Index of Occupational Status.

To what extent are personality scores concurrently associated with grades (M1), even beyond subject-specific and general cognitive competence scores (M2) and sociodemographic covariates (M3)? To address this question, we analyzed models M1–M3. Figure 2 displays standardized regression coefficients and 95% confidence intervals; parameter estimates and further model details are shown in Table S2. To allow for comparisons of effect sizes, we also display the coefficients of the competence scores and covariates. 

Our results allow for some general observations to be made: First, the increasingly comprehensive models explained increasing amounts of variance (e.g., for German in the 7th grade, for M1, M2, and M3, *R*^2^ = .06, .24, and .26, respectively; see also Table S2), suggesting that each set of predictors—personality, competencies, and sociodemographic covariates—was uniquely related to school grades. Averaged across subjects and grade levels, personality explained 4.4% of the variance in grades; this average share of explained variance decreased only slightly to 3.7% when subject-specific and general competencies and covariates were controlled for (see Table S2). Second, the pattern of results was largely similar for the 7th and 9th graders, suggesting only small differences in personality–grade associations across grade levels.^5^ Third, the pattern of results was similar for mathematics and science but differed for German, which points toward similarities and differences in grading practices across subjects. 

Focusing on the dimension-specific associations between personality and grades, conscientiousness was consistently the strongest predictor of grades. The standardized competence- and covariate-controlled estimates of conscientiousness for grades (M3) were 2.2 to 6.4 times larger than the estimates of the respective second-largest dimension. As expected, conscientiousness was positively related to teacher-assigned grades across all three subjects and timepoints (e.g., M1 for German in 9th grade: β = 0.19, SE = .01, 95% CI [0.16, 0.22]), and these relations remained very similar after controlling for competencies (e.g., M2 for German in 9th grade: β = 0.19, SE = .01, 95% CI [0.17, 0.22]) and covariates (e.g., M3 for German in 9th grade: β = 0.17, SE = .01, 95% CI [0.15, 0.20]). 

For openness, the results were more subject-specific and generally showed the greatest discrepancies when competencies were versus were not controlled for. For example, the standardized coefficients for openness were on average reduced by 0.044 when competencies were controlled for (i.e., ΔM1 M2), whereas for conscientiousness this average absolute discrepancy was 0.019. For German, openness was positively related with grades both in 7th and 9th grade (e.g., M1 in 7th grade: β = 0.10, SE = .01, 95% CI [0.07, 0.12]). Controlling for competencies and covariates, relations were substantially reduced and in 7th grade no longer reached statistical significance. Unexpectedly, for mathematics and science, openness showed null relations with grades. Controlling for competencies and covariates further diminished these relations, such that openness was even negatively related with grades (e.g., M3 for mathematics in 7th grade: β = −0.05, SE = .01, 95% CI [−0.08, −0.03]).^6^

Regarding the remaining personality domains, for neuroticism, negative associations with mathematics and science grades were found (e.g., M1 for mathematics in 7th grade: β = −0.06, SE = .01, 95% CI [−0.08, −0.03]). When competencies and covariates were controlled for, these associations vanished in 7th grade but stayed significant in 9th grade. For extraversion, positive associations with German grades were found, which remained after controlling for competencies and covariates both in 7th and 9th grade (e.g., M3 in 9th grade: β = 0.06, SE = .01, 95% CI [0.03, 0.09]). For math and science, extraversion was not significantly related to grades in 7th grade. However, in 9th grade, more extraverted students received lower mathematics grades, also after controlling for competencies and covariates (M3 in 9th grade: β = −0.05, SE = .01, 95% CI [−0.08, −0.02]). Agreeableness was not significantly related to teacher-assigned grades across any model, subject, or grade level. 

### 3.2. Longitudinal Analyses 

**Figure 3:**
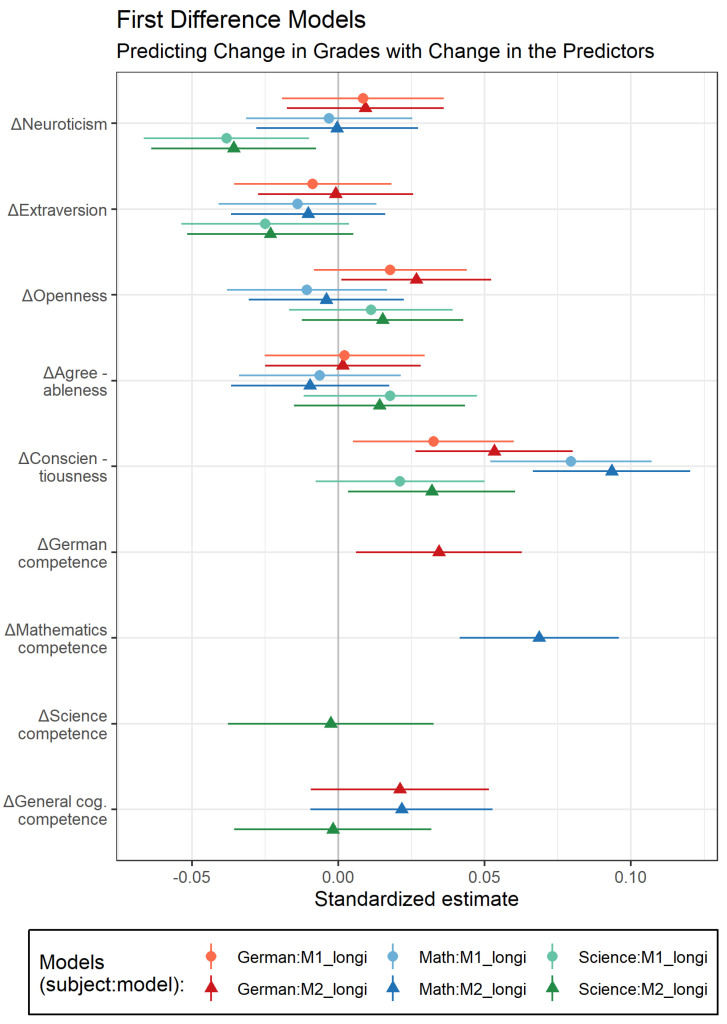
Standardized estimates for the longitudinal models.

Are individual differences in change in personality related to individual differences in change in grades (M1_longi_), even beyond individual differences in change in competence test scores (M2_longi_)? To answer this question, we analyzed the first-difference models M1_longi_ and M2_longi_. Standardized regression coefficients and 95% confidence intervals are displayed in Figure 3; parameter estimates and further model details are given in Table S4.

The longitudinal associations between personality and grades were generally smaller than the concurrent associations. As expected, change in conscientiousness was positively related to change in grades across all three subjects and when controlling for change in competencies (e.g., M2_longi_ for German: β = 0.05, SE = .01, 95% CI [0.03, 0.08])^7^. Similar to the cross-sectional results, for change in openness, a positive association with change in German grades emerged. Students who became more open over time improved their German grades, but only when change in competencies was controlled for (M2_longi_ for German: β = 0.03, SE = .01, 95% CI [0.001, 0.05]). Of the remaining three personality dimensions, longitudinal effects were observed only for neuroticism. Persons who became more neurotic obtained lower science grades over time, even beyond change in science competencies (M2_longi_: β = −0.04, SE = .014, 95% CI [−0.07, −0.01]). 

Overall, although some coefficients for personality change differed in their significance upon considering competence change, these differences were only small in magnitude. This suggests that the relations between change in personality and change in grades were largely independent of change in competencies.

### 3.3. Robustness Checks 

We conducted three robustness checks. First, we explored whether the competence-independent associations between personality and grades existed across school types. School type has been shown to moderate the associations between personality and grades (Brandt et al. 2020; Poropat 2009; Tetzner et al. 2020), suggesting that personality unfolds differently in different learning environments. We therefore explored the cross-sectional competence- and covariate-controlled relations between personality and grades (i.e., M3) with multigroup path models separately for students in academic-track and non-academic-track schools. For all models, constraining regression coefficients to equality across school types yielded a significantly worse model fit. In line with previous findings (Brandt et al. 2020), the associations between personality and grades were stronger for academic-track schools than for non-academic-track schools, and this tendency was more evident in 9th grade (see Table S5 and Figure S1). Other results were shown to be robust across academic- and non-academic-track schools. For example, for all 30 (pairs of) regression coefficients for personality on grades, the 95% CI overlapped in the models for academic- and non-academic-track schools. Moreover, for all models, competence-independent associations of conscientiousness with grades were observed across both school types. Hence, competence- and covariate-controlled associations of personality with grades emerged regardless of school type. 

Second, we considered potential referential biases in grading practices (“grading on a curve”, e.g., Calsamiglia and Loviglio 2019). We therefore centered the grades within classes (if *n* ≥ 5 students provided data within that class) and re-ran the cross-sectional analyses (see Table S6 and Figure S2). Overall, centering grades did not substantially alter the pattern of results. The contributions of personality were highly similar to the uncentered results (e.g., Δ*R*^2^ between M1 and M1_centered_ ranged between −.004 and .008), suggesting that the personality–grade relations were only marginally influenced by referential grading. 

Third, claims about incremental validity are associated with high rates of false positive results (also known as residual confounding; Westfall and Yarkoni 2016). To overcome potentially spurious results, measurement unreliability should be considered (Westfall and Yarkoni 2016). We followed this recommendation and reanalyzed the cross-sectional models, modeling the scores’ unreliability with single-indicator structural equation models. The results (posted on the OSF) remained highly similar, providing further evidence for the incremental contributions of personality to grades above and beyond competencies in those subjects.

## 4. Discussion 

Do the associations between personality and school grades reflect the mastery of competencies in the taught subjects? Or are they independent of competencies and reflect other factors such as teachers rewarding desirable personality traits in their grading? In the current study, we triangulated different analyses and examined the associations of the Big Five with German, mathematics, and science grades while controlling for subject-specific and general cognitive competencies and sociodemographic covariates. 

Results revealed that the predictive power of personality for grades was largely independent of competencies. The largest competence-independent contributions emerged for conscientiousness, also from a longitudinal perspective. This suggests that personality–grade associations in general, and conscientiousness–grade associations in particular, reflect mechanisms other than actual competence in the course content. For openness, we found a slightly different pattern, where the associations with grades revealed more discrepancies when competencies were controlled for versus were not controlled for. Illustrating parallels between openness and cognitive abilities (DeYoung et al. 2014; Ziegler et al. 2012), this suggests that the openness–grade associations may also operate through competence gains. 

The results challenge an implicit assumption that seems to underlie much research on personality–achievement associations—namely, that these associations primarily reflect the potential of personality to foster learning and competence mastery. Together with findings showing that associations of personality with grades are typically greater than those with competence tests (e.g., Borghans et al. 2016; Brandt et al. 2020; Lechner et al. 2017; Spengler et al. 2013), our results suggest that personality–grade associations reflect mechanisms other than competence mastery, such as possibly teachers’ grading practices (Poropat 2014b; Westphal et al. 2021; Willingham et al. 2002). Although it is beyond the scope of the present study to explicate the mechanisms that might underlie the personality–grade associations in detail, our results can inform hypotheses about these mechanisms.

### 4.1. How Does the Predictive Power of Personality for School Grades Unfold? 

#### 4.1.1. Personality Has Largely Competence-Independent Associations with Grades

Our results show that the associations of personality with grades were largely independent of competencies in the subjects in question. Even when accounting for other key predictors—subject-specific and general cognitive competencies, sociodemographic covariates—personality incrementally predicted variance in teacher-assigned grades. Four main results can give insights into the nature of these competence-independent contributions of personality. 

First, our results corroborate previous findings about the outstanding role of conscientiousness for grades (Dumfart and Neubauer 2016; Mammadov 2021; Poropat 2009, 2014b). What is more, conscientiousness–grade associations were largely independent of subject-specific and general cognitive competencies. Hence, the power of conscientiousness in the classroom appears to unfold almost entirely independently of course mastery and general cognitive competence (see also Spengler et al. 2013; Westphal et al. 2021). 

Second, for the remaining personality dimensions, competence-independent relations were more subject-specific. If, for example, two 9th graders differed in their extraversion but not in their German or mathematics competence (or any other predictors), on average, the more extraverted student received higher grades in German but lower grades in mathematics. Thus, specific behavioral tendencies—holding competencies constant—can be rewarded with better grades in only specific subjects (see also Brandt et al. 2020). 

Third, although they were smaller than the cross-sectional relations, we also found longitudinal competence-independent contributions of personality to grades (see also Israel et al. 2019; Spengler et al. 2016; Noftle and Robins 2007). Again, the strongest effects emerged for conscientiousness. If students became more conscientious between 7th and 9th grade, on average, their grades improved—regardless of competence change. The analyzed first-difference models account for any (omitted) time-invariant covariates (Liker et al. 1985), such as sex or stable genetic differences. Thus, these models rigorously tested the relevance of personality change and underlined the prospective importance of conscientiousness for grades. Furthermore, the results also suggest that longitudinal effects of conscientiousness do not unfold through change in competencies. 

Fourth, the competence-independent contributions of personality showed only minor differences across grade levels (7th vs. 9th grade). However, in line with earlier findings (e.g., Andersen et al. 2020; Tetzner et al. 2020), our results also revealed small differences across grade levels. For example, whereas competence-independent extraversion was unrelated to mathematics grades in 7th grade, it showed negative relations in 9th grade. In a similar vein, while the competence-independent contributions of personality were also generally comparable across academic and non-academic schools, there was a tendency for stronger relations in academic schools. Overall, whether students were at an earlier or a more advanced stage in their secondary school studies, or at academic or non-academic schools, certain behavioral patterns appeared to be similarly associated with grading.

#### 4.1.2. Competence-Related Associations Can Emerge for Openness 

Although most associations of personality with grades appeared to be largely independent of competencies, our results also suggest that personality–grade associations might also reflect gains in competencies. Controlling for subject-specific and general cognitive competencies influenced the results for openness. This suggests that seeking out and intellectually or creatively engaging with new input may facilitate knowledge acquisition, increase competence (Hübner et al. 2022), and thus be associated with better grades. These mechanisms are consistent with theorizing about the nature of openness (von Stumm 2018; Ziegler et al. 2018). Moreover, our data suggest that this competence-facilitating potential of openness differs across subjects (see also, Brandt et al. 2020). Particularly for German, the openness–grade association overlapped with competencies, indicating that an open student’s good grades in that subject are indeed linked to their linguistic mastery of German. For mathematics and science, openness–grade associations were also affected when controlling for competencies. However, the competence-independent effects of openness then even had negative associations with grades. This might be due to the construct space of openness. The BFI-10 focuses on creative, aesthetic aspects of openness (Rammstedt and John 2007). Potentially, such creative aspects—when accounted for competencies—might hinder the achievement of good grades in mathematics or physics, where adherence to mathematical rules may be critical to success. Consistent with this reasoning, in a qualitatively informed survey, prospective mathematics and science teachers were shown to consider unique and creative student answers a source of distraction more commonly than teachers of social science subjects did (Beghetto 2007). 

### 4.2. What Do Competence-Independent Associations between Personality and Grades Reflect?

Our finding that personality–grade associations were largely independent of competencies allows us to speculate about why personality is related to grades—if not via competence-related mechanisms. Many of the factors invoked in prior research to explain personality–achievement associations—such as learning strategies and behavior, willingness to perform, and focus on learning tasks (De Raad and Schouwenburg 1996; Saklofske et al. 2012; Zhang and Ziegler 2016)—should lead to competence gains and thus also be reflected in standardized achievement test scores. Therefore, such mechanisms are unlikely to explain the competence-independent contributions of personality to grades. 

If specific behavioral tendencies do not pay off through competence-facilitation, they must operate “non-cognitively” instead. Competence-independent associations could plausibly be explained through teachers’ grading practices (Westphal et al. 2021; Willingham et al. 2002). Building on expectations about what a good student should be like, teachers may reward desirable behaviors and traits, regardless of whether these traits relate to higher subject-specific competencies. Indeed, the clear competence-independent contribution of conscientiousness aligns with earlier findings showing that teachers consider this trait to be beneficial in learning contexts (e.g., Barbaranelli et al. 2003; Poropat 2014b). Such grading practices might reflect deliberate attempts to shape students’ personalities in desirable ways. Teachers might intentionally reward these traits to reinforce traits assumed to be beneficial for competence development. Alternatively, such practices might also reflect a halo effect (Thorndike 1920). Positive assumptions about specific behaviors might imply a halo that inadvertently affects teachers’ grading (Poropat 2014a). 

Our results suggest that these grading practices are by no means the preferences of individual teachers. The competence-independent contribution of conscientiousness was shown for German, mathematics, and science; across 7th and 9th grade; across school types; and across a very likely change of teachers over the years. Thus, teachers generally seem to reward conscientiousness, even if conscientious students are not in fact more competent. Our results also indicated a tendency for slightly stronger competence-independent personality contributions in academic track schools. Potentially, different classroom demands or achievement-related norms across school types might reward trait expressions differently (see also Brandt et al. 2020; Tetzner et al. 2020). 

Additionally, subject-specific results for extraversion, neuroticism, and openness suggest that personality–grade associations might also reflect subject-specific classroom requirements. While, for example, in German, successful classroom behavior may require extraverted engagement in class discussions, in mathematics, it may be based on precise answers or independent task completion (see also Brandt et al. 2020). Importantly, these results suggest that such subject-specific classroom requirements are also independent of subject-specific competencies, meaning that, for instance, merely participating in the discussion would be rewarded, not the contribution’s quality. Additionally, these subject-specific relations for (non-cognitive) classroom behaviors could potentially be related to differences in teacher characteristics across subject domains (e.g., Hartmann and Ertl 2021; see also Beghetto 2007). 

#### Zooming Further into the Personality–Grade Associations

To better understand how the power of personality unfolds in the classroom, at least four directions for further study can be identified. First, the behavioral consequences of personality should be explicitly assessed. Along with cross-sectional studies that focus on theory-derived behavioral mediators (e.g., Steinmayr et al. 2011), experience sampling studies in which students repeatedly provide information on their behavior during class might prove useful (see Trautwein et al. 2009). Second, the hypothesis that the competence-independent contributions of personality might reflect grading practices or halo effects should be tested. Here, teachers’ perceptions of the “ideal student” (Kitchin 1972) or the incremental value of teachers’ reports on students’ personalities beyond the students’ self-reports seem particularly important (Brandt et al. 2021; Poropat 2014b). Third, differential effects by gender should be explored in greater detail. Gender-related competence differences (e.g., Else-Quest et al. 2010; Reilly et al. 2019) and teachers’ beliefs (e.g., Li 1999) could lead to insightful gender-specificities in the competence-independent contributions of personality. Fourth, a deeper understanding of the criterion variable—grades—would be useful. Our results suggest that grades can be subsumed under a broad understanding of competence (Blömeke et al. 2015), such that grades reflect cognitive abilities and the ability to show relevant behavior in the required situations (Willingham et al. 2002). However, our data suggest that grades can also reflect additional information. Hence, the construct space of grades should be carefully delineated. 

### 4.3. Limitations and Directions for Future Research

Despite featuring strengths such as a large and representative dataset and a comprehensive exploration of personality–grade associations, several limitations of the present study should be considered. First, although the competence tests provided in NEPS were designed to meet German national educational standards (e.g., Hahn et al. 2013; Neumann et al. 2013), they may not directly correspond to the subject-specific competencies that teachers grade. For example, the mathematics competence tests might be limited to problem-based questions and not cover all math-related competencies graded by teachers, such as proficiency in using mathematical tools such as calculators (Neumann et al. 2013). Controlling for competence scores might thus not fully account for the total competence-related variance in personality–grade associations. This might have decreased the evidence for competence-related relations and increased it for competence-independent relations. We sought to address this limitation by additionally accounting for general cognitive abilities in the models. Nonetheless, future research would do well to replicate and extend our findings with competence measures that vary in their degree of curricular validity. 

Similarly, the matrices test (Haberkorn and Pohl 2013) that we used as an indicator for general cognitive competencies might be deemed to be a narrow conceptualization. Although matrices tests are considered to be among the best indicators for fluid intelligence (Nisbett et al. 2012), a single measure nonetheless captures a limited picture of the cognitive processes subsumed under general cognitive competencies (Schneider and McGrew 2018). 

Next, different facets of the same personality domains have been shown to have differential associations with grades (Noftle and Robins 2007); however, our short personality measure did not allow us to examine facet-level associations. Relatedly, the relatively narrow assessment of the Big Five and the comparably low reliability estimates of their scale scores might have led to an underestimation of the predictive power of personality. Such an underestimation could have affected the estimation of both the competence-related and competence-independent relations. When considering the imperfect measurement reliability in our robustness check (see Westfall and Yarkoni 2016), however, the overall pattern of results remained similar, further supporting the incremental effects of personality on grades. Yet, future work should use more comprehensive personality measures and explicitly consider Big Five facets or nuances (Bipp et al. 2008; Kretzschmar et al. 2018; Rozgonjuk et al. 2021). Such data could further inform the mechanisms underlying the personality–grade associations.

Data from the earlier waves of the current study are now aged by about a decade. While the associations between personality and competencies have been robustly shown across data from different periods (e.g., Ackerman 1996; Mammadov 2021; Poropat 2009), the personality–grade associations might differ as a function of period-specific educational reforms or grading practices. To explore such potential period-specific effects, personality–grade relations should be examined together with data on educational policies. 

Lastly, in the longitudinal analyses, we used first-difference models (e.g., Liker et al. 1985) to explore whether change in personality and change in competencies were related to change in grades. Such models have the advantage of differencing out any time-invariant, unmeasured confounders and time-consistent measurement biases (Liker et al. 1985). However, the use of change scores has been argued to be problematic due to their unreliability (e.g., Cronbach and Furby 1970; however, see Rogosa and Willett 1983). The current results may thus rather conservatively estimate the relations between personality change and grade change. 

## 5. Conclusions

The current study furthers the understanding of how the power of personality unfolds in the classroom. Personality predicted teacher-assigned grades, and it did so largely independently of students’ competencies in the subjects in question. These results challenge the assumption that personality–grade associations result from learning processes and competence acquisition and suggest that personality–grade associations predominantly reflect the socioemotional value of personality as rewarded in teachers’ grading practices. As such, the results encourage a more fine-grained view of the interplay between personality, subject-specific and general cognitive competencies, classroom behavior, and grades. 

## Data Availability

The NEPS data are available to individuals with a valid NEPS data usage contract. The preregistration, analysis scripts, and additional material can be found on OSF: https://osf.io/vmn9p/.

## Notes

1 The percentage of missing data in personality and competence variables ranged between 16% (for conscientiousness) and 38% (scientific competencies) in 7th grade and between 29% (openness) and 42% (general cognitive competencies) in 9th grade. For teacher-assigned grades, the range of the proportion of missing data was between 23% (German grades in 7th grade) and 37% (physics grades in 9th grade). For the covariates, the proportion of missing data ranged between 1% (gender) and 33% (parental socioeconomic status).

2 Up to four science subjects—physics, chemistry, biology, and natural science—are taught at German secondary schools. Subjects vary across schools and federal states. Of those four subjects, physics was reported most often in the current data. We therefore present the results for physics grades, with the remaining three subject grades used as auxiliary variables (Enders 2008). A composite grade across all science subjects might blur potential evidence for teacher-specific grading practices. However, as preregistered, we also report the results for a composite grade across all science subjects on the OSF. The interpretation of the results remains highly similar. This suggests that the results reported for physics grades similarly apply to other science subjects.

3 https://osf.io/vmn9p/ (last accessed on 19 April 2022). We also share the analysis code, output, and additional analyses in this OSF project.

4 For example, for extraversion, the pseudo-latent difference variable was defined by extraversion in 9th grade as a single indicator; the loading was fixed to 1. Additionally, extraversion in 9th grade was regressed on extraversion in 7th grade. Restricting the residual variance of extraversion in 9th grade to zero, the pseudo-latent difference variable represented the difference between extraversion in 7th and 9th grade.

5 For science, the assessment schedule differed from that used for German and mathematics. For younger students (i.e., personality assessment in 7th grade), science competence was assessed in 6th grade, two years before the science grades were given in 8th grade. This could have decreased the overlap between the science competence scores and grades. For older students, science competence and science grades were both assessed in 9th grade. The results were largely similar to those obtained for younger students, suggesting that there was no bias due to the assessment schedule.

6 These results suggest that the relations between openness and grades might be mediated by competencies. To further inform this interpretation, we analyzed the most comprehensive models M3 within a mediation framework; results are reported in Table S3. Openness was the only dimension that consistently showed mediation effects via subject-specific competencies across all subjects and grade levels. Since these analyses are based on cross-sectional data, causal interpretation is not warranted.

7 For science, change in conscientiousness predicted change in grades only when change in competencies was controlled for (M1_longi_: β = 0.02, SE = .015, 95% CI [−0.01, 0.05]; M2_longi_: β = 0.03, SE = .015, 95% CI [0.003, 0.06]).

## Supplementary Materials

The following supporting information can be downloaded at: https://www.mdpi.com/article/10.3390/jintelligence10020026/s1: Table S1: Means, Standard Deviations, and Correlations Among the Measured Variables; Table S2: Standardized Regression Coefficients for the Cross-sectional Models Predicting German, Math, and Science Grades; Table S3: Standardized Coefficients from the Cross-sectional Mediation Analyses Predicting German, Mathematics, and Science Grades via Subject-specific Competencies; Table S4: Standardized Estimates for the Longitudinal Models Predicting Change in Grades with Change in Personality and Competencies; Table S5: Standardized Regression Coefficients for the Cross-sectional Models Predicting German, Math, and Science Grades; Separated for Students at Non-Academic and Academic Track Schools; Table S6: Class-centered, Standardized Regression Coefficients for the Cross-sectional Models Predicting German, Math, and Science Grades; Figure S1: Standardized Estimates for the Cross-Sectional Models, Separated for Students at Non-Academic and Academic Track Schools; Figure S2: Standardized Estimates for the Cross-Sectional Models, Grades Centered Within Class.
