# Supplementary material for: Beyond Competencies: Associations between Personality and School Grades Are Largely Independent of Subject-Specific and General Cognitive Competencies
Source: J Intell. 2022 Apr 27;10(2):26. doi: 10.3390/jintelligence10020026 (PMC9149965; doi:10.3390/jintelligence10020026)
Supplement: Supplementary file 1 [file jintelligence-10-00026-s001.zip › jintelligence-1593035-supplementary.pdf]

Table S1

Means, Standard Deviations, and Correlations Among the Measured Variables

Variable	*M*	*SD*	1	2	3	4	5	6	7	8	9	10	11	12	13	14	15	16	17	18	19	20	21	22	23	24	25	26
7th Grade																												
1. Neuroticism	2.83	0.83																										
2. Extraversion	3.40	0.79	-.23*																									
3. Openness	3.45	0.96	.02	.09*																								
4. Agreeableness	3.46	0.65	-.02	-.01	.19*																							
5. Conscientious.	3.23	0.85	-.04*	.00	.10*	.30*																						
6. General Cog. Comp[a]	6.89	2.62	-.06*	-.00	.09*	-.01	-.04																					
7. German Comp.	-0.00	0.89	-.02	.06*	.15*	.05*	.09*	.44*																				
8. Math Comp.	0.72	1.22	-.10*	.02	.09*	-.01	-.01	.54*	.63*																			
9. Science Comp.[b]	0.08	1.15	-.10*	.02	.11*	-.04*	-.05*	.47*	.62*	.66*																		
10. German Grade	4.30	0.83	-.01	.09*	.13*	.08*	.20*	.17*	.45*	.27*	.29*																	
11. Math Grade	4.21	0.98	-.06*	-.01	.00	.03	.18*	.27*	.26*	.38*	.27*	.47*																
12. Science Grade[c]	4.26	0.97	-.04*	.01	.03	.04*	.16*	.17*	.21*	.28*	.25*	.36*	.48*															
9th Grade																												
13. Neuroticism	2.85	0.86	.37*	-.12*	.06*	.04*	.00	-.06*	.01	-.10*	-.08*	.03	-.07*	-.05*														
14. Extraversion	3.30	0.85	-.19*	.48*	.03*	-.01	.02	-.04*	-.01	-.01	-.02	.07*	-.01	.01	-.29*													
15. Openness	3.37	0.95	-.01	.05*	.52*	.11*	.05*	.06*	.10*	.05*	.08*	.10*	-.00	.03	.03	.05*												
16. Agreeableness	3.44	0.65	.04*	-.01	.09*	.38*	.19*	-.00	.01	-.05*	-.05*	.03	.01	.02	-.01	-.01	.13*											
17. Conscientious.	3.05	0.83	-.03	.00	.05*	.17*	.51*	-.11*	-.03*	-.10*	-.11*	.16*	.13*	.20*	-.01	.07*	.08*	.19*										
18. gf	9.20	2.25	-.05*	-.02	.10*	.03	.01	.50*	.43*	.51*	.44*	.17*	.25*	.18*	-.03	-.03	.09*	.03	-.05*									
19. German Comp.	-0.00	0.91	-.02	.04*	.16*	.06*	.10*	.42*	.80*	.56*	.59*	.42*	.25*	.23*	.03	-.03	.11*	.02	.01	.47*								
20. Math Comp.	0.01	1.19	-.07*	-.01	.06*	-.04	.01	.49*	.60*	.74*	.65*	.29*	.41*	.33*	-.07*	-.04*	.03	-.05*	-.05*	.53*	.61*							
21. Science Comp.	0.04	0.93	-.08*	-.00	.13*	-.04*	-.02	.45*	.61*	.65*	.68*	.28*	.31*	.30*	-.07*	-.05*	.12*	-.05*	-.09*	.45*	.61*	.70*						
22. German Grade	4.24	0.84	-.00	.09*	.14*	.08*	.19*	.15*	.38*	.22*	.25*	.54*	.31*	.35*	.03	.07*	.14*	.06*	.21*	.15*	.38*	.25*	.23*					
23. Math Grade	4.10	1.03	-.04*	-.04*	-.01	.04	.13*	.22*	.21*	.32*	.23*	.33*	.56*	.50*	-.05*	-.04	-.01	.02	.18*	.24*	.22*	.38*	.28*	.40*				
24. Science Grade	4.21	1.00	-.02	.01	.00	.03	.14*	.19*	.18*	.24*	.23*	.31*	.42*	.62*	-.07*	.00	.02	.03	.18*	.18*	.22*	.30*	.27*	.39*	.54*			
Covariates																												
25. Migrat. Status	0.15	0.36	.00	-.02	.00	-.00	.02	-.13*	-.14*	-.16*	-.26*	-.11*	-.09*	-.07*	.01	.01	-.01	.01	.02	-.12*	-.12*	-.15*	-.19*	-.10*	-.04*	-.06*		
26. HISEI	-0.00	1.00	-.06*	.08*	.09*	.04*	.04	.22*	.35*	.36*	.35*	.18*	.16*	.17*	-.03	.05*	.06*	.01	-.02	.24*	.35*	.37*	.35*	.20*	.17*	.14*	-.19*	
27. Gender	0.48	0.50	.16*	.04*	.22*	.18*	.18*	-.04*	.16*	-.14*	-.09*	.21*	-.04*	-.01	.26*	-.02	.21*	.13*	.16*	.02	.19*	-.12*	-.06*	.26*	-.01	-.01	-.00	.00

Note. cog. = cognitive; Comp. = Competence; HISEI = highest socioeconomic status.

[a] assessed in 5th grade; [b] assessed in 6th grade; [c] referring to 8th grade.

* $p < .01$.

Table S2. *Standardized Regression Coefficients for the Cross-sectional Models Predicting German, Math, and Science Grades*

	7th Grade									9th Grade								
	Model 1			Model 2			Model 3			Model 1			Model 2			Model 3		
	β	SE	*p*	*β*	SE	*p*	*β*	SE	*p*	*β*	SE	*p*	*β*	SE	*p*	*β*	SE	*p*
German																		
Neuroticism	0.007	0.013	.591	0.015	0.012	.224	-0.006	0.012	.656	0.044	0.015	.002	0.040	0.014	.003	-0.001	0.014	.926
Extraversion	0.086	0.014	<.001	0.067	0.013	<.001	0.058	0.012	<.001	0.060	0.015	<.001	0.069	0.014	<.001	0.059	0.014	<.001
Openness	0.098	0.013	<.001	0.037	0.013	.003	0.019	0.013	.142	0.123	0.014	<.001	0.078	0.013	<.001	0.049	0.013	<.001
Agreeableness	0.001	0.015	.954	-0.003	0.013	.799	-0.018	0.013	.171	0.008	0.015	.602	0.007	0.014	.621	-0.007	0.014	.622
Conscientiousness	0.189	0.014	<.001	0.157	0.013	<.001	0.144	0.013	<.001	0.190	0.014	<.001	0.191	0.013	<.001	0.174	0.013	<.001
German Comp.				0.438	0.014	<.001	0.400	0.015	<.001				0.384	0.015	<.001	0.322	0.017	<.001
General Cog. Comp.				-0.010	0.016	.517	-0.004	0.016	.812				-0.018	0.016	.278	-0.017	0.016	.310
Migration Status							-0.054	0.013	<.001							-0.051	0.014	<.001
HISEI							0.035	0.015	.021							0.079	0.016	<.001
Gender							0.119	0.012	<.001							0.167	0.013	<.001
R^2		.058			.240			.256			.062			.203			.234	
$\Delta R^2_{Big\ Five}$[a]					.024									.038				
Math																		
Neuroticism	-0.057	0.014	<.001	-0.013	0.013	.306	-0.013	0.013	.320	-0.070	0.015	<.001	-0.032	0.014	.023	-0.038	0.015	.010
Extraversion	-0.023	0.014	.092	-0.015	0.013	.245	-0.016	0.013	.192	-0.070	0.015	<.001	-0.045	0.014	.002	-0.048	0.014	.001
Openness	-0.007	0.013	.596	-0.052	0.012	<.001	-0.052	0.013	<.001	-0.017	0.014	.249	-0.044	0.013	.001	-0.049	0.014	<.001
Agreeableness	-0.028	0.014	.046	-0.017	0.013	.195	-0.018	0.013	.176	-0.012	0.015	.413	0.004	0.014	.796	0.001	0.014	.929
Conscientiousness	0.186	0.014	<.001	0.198	0.013	<.001	0.198	0.013	<.001	0.184	0.014	<.001	0.207	0.013	<.001	0.205	0.014	<.001
Math Comp.				0.333	0.015	<.001	0.322	0.017	<.001				0.355	0.016	<.001	0.351	0.017	<.001
General Cog. Comp.				0.112	0.018	<.001	0.110	0.018	<.001				0.063	0.017	<.001	0.061	0.017	<.001
Migration Status							-0.021	0.014	.124							0.028	0.014	.044
HISEI							0.022	0.016	.174							0.031	0.017	.064
Gender							-0.003	0.013	.825							0.021	0.014	.132
R^2		.036			.196			.197			.039			.191			.192	
$\Delta R^2_{Big\ Five}$[a]					.039									.044				
Science																		
Neuroticism	-0.039	0.015	.009	-0.007	0.015	.628	-0.004	0.015	.771	-0.072	0.015	<.001	-0.041	0.015	.005	-0.039	0.015	.012
Extraversion	0.003	0.015	.836	0.011	0.014	.450	0.005	0.014	.722	-0.029	0.015	.052	-0.008	0.014	.595	-0.010	0.014	.499
Openness	0.013	0.014	.351	-0.030	0.014	.042	-0.028	0.015	.057	0.006	0.015	.698	-0.041	0.014	.005	-0.039	0.015	.009
Agreeableness	-0.010	0.015	.510	0.007	0.015	.636	0.005	0.015	.765	-0.009	0.015	.571	0.004	0.015	.812	0.004	0.015	.797
Conscientiousness	0.165	0.015	<.001	0.180	0.014	<.001	0.178	0.014	<.001	0.184	0.015	<.001	0.214	0.014	<.001	0.215	0.014	<.001
Science Comp.				0.227	0.018	<.001	0.196	0.020	<.001				0.259	0.017	<.001	0.245	0.018	<.001
General Cog. Comp.				0.088	0.018	<.001	0.081	0.018	<.001				0.078	0.017	<.001	0.075	0.017	<.001
Migration Status							-0.004	0.016	.818							-0.006	0.015	.698
HISEI							0.086	0.018	<.001							0.037	0.018	.042
Gender							-0.016	0.014	.280							-0.012	0.014	.397
R^2		.029			.104			.111			.038			.126			.127	
$\Delta R^2_{Big\ Five}$[a]					.032									.045				

Note. [a] Incremental contributions of the Big Five beyond competencies and covariates (i.e., ΔR^2 between Model M3 and a model with all predictors except the Big Five).

Table S3. *Standardized Coefficients from the Cross-sectional Mediation Analyses Predicting German, Mathematics, and Science Grades via Subject-specific Competencies*

	German			Mathematics			Science		
	β	SE	*p*	*β*	SE	*p*	*β*	SE	*p*
7th Grade									
Direct Relations: Personality → Competence									
N	-0.015	0.013	.244	-0.104	0.013	<.001	-0.121	0.017	<.001
E	0.047	0.013	<.001	-0.008	0.013	.522	-0.016	0.017	.337
O	0.146	0.013	<.001	0.102	0.013	<.001	0.143	0.016	<.001
A	0.005	0.014	.728	-0.029	0.014	.037	-0.060	0.018	.001
C	0.075	0.014	<.001	-0.019	0.014	.192	-0.056	0.017	.001
Direct Relation: Competence → Grade									
Comp	0.398	0.013	<.001	0.343	0.014	<.001	0.197	0.019	<.001
Direct Relations: Personality → Grade									
N	-0.005	0.012	.689	-0.010	0.013	.425	0.000	0.015	.991
E	0.059	0.013	<.001	-0.016	0.012	.204	0.004	0.014	.792
O	0.016	0.013	.215	-0.057	0.013	<.001	-0.030	0.015	.040
A	-0.018	0.014	.182	-0.017	0.014	.203	0.006	0.016	.707
C	0.146	0.012	<.001	0.200	0.013	<.001	0.181	0.015	<.001
Indirect Relations: Personality → Grade									
N	-0.006	0.005	.244	-0.036	0.005	<.001	-0.024	0.004	<.001
E	0.019	0.005	<.001	-0.003	0.004	.522	-0.003	0.003	.338
O	0.058	0.006	<.001	0.035	0.005	<.001	0.028	0.004	<.001
A	0.002	0.006	.728	-0.010	0.005	.037	-0.012	0.004	.001
C	0.030	0.006	<.001	-0.006	0.005	.193	-0.011	0.004	.002
9th Grade									
Direct Relations: Personality → Competence									
N	0.016	0.015	.306	-0.096	0.015	<.001	-0.100	0.015	<.001
E	-0.030	0.016	.052	-0.068	0.015	<.001	-0.074	0.015	<.001
O	0.125	0.015	<.001	0.054	0.014	<.001	0.145	0.014	<.001
A	0.010	0.016	.536	-0.043	0.015	.005	-0.045	0.015	.002
C	0.001	0.015	.964	-0.047	0.014	.001	-0.095	0.014	<.001
Direct Relation: Competence → Grade									
Comp	0.329	0.016	<.001	0.346	0.016	<.001	0.244	0.017	<.001
Direct Relations: Personality → Grade									
N	-0.002	0.014	.877	-0.037	0.015	.015	-0.038	0.016	.017
E	0.060	0.014	<.001	-0.048	0.014	.001	-0.010	0.015	.509
O	0.047	0.014	.001	-0.050	0.013	<.001	-0.040	0.015	.008
A	-0.007	0.014	.623	0.002	0.014	.870	0.005	0.015	.748
C	0.176	0.013	<.001	0.208	0.014	<.001	0.218	0.015	<.001
Indirect Relations: Personality → Grade									
N	0.005	0.005	.306	-0.033	0.006	<.001	-0.024	0.004	<.001
E	-0.010	0.005	.052	-0.024	0.005	<.001	-0.018	0.004	<.001
O	0.041	0.005	<.001	0.019	0.005	<.001	0.035	0.004	<.001
A	0.003	0.005	.537	-0.015	0.005	.006	-0.011	0.004	.003
C	0.000	0.005	.964	-0.016	0.005	.001	-0.023	0.004	<.001

Note. N = neuroticism; E = extraversion; O = openness; A = agreeableness; C = conscientiousness; Comp = subject-specific competence.

The mediation analyses build on the cross-sectional models M3. Displayed are only the parameters related to the mediation effect of personality. The cross-sectional nature of the data does not warrant causal interpretation.

Table S4

Standardized Estimates for the Longitudinal Models Predicting Change in Grades with Change in Personality and Competencies

	Change in German Grades						Change in Math Grades						Change in Science Grades					
	Model 1 $_{longi}$			Model 2 $_{longi}$			Model 1 $_{longi}$			Model 2 $_{longi}$			Model 1 $_{longi}$			Model 2 $_{longi}$		
	β	SE	*p*	*β*	SE	*p*	*β*	SE	*p*	*β*	SE	*p*	*β*	SE	*p*	*β*	SE	*p*
Change in Neuroticism	0.008	0.014	.549	0.009	0.014	.500	-0.003	0.014	.829	0.000	0.014	.977	-0.038	0.014	.008	-0.036	0.014	.013
Change in Extraversion	-0.009	0.014	.527	-0.001	0.014	.946	-0.014	0.014	.312	-0.010	0.013	.444	-0.025	0.015	.087	-0.023	0.014	.109
Change in Openness	0.018	0.013	.183	0.027	0.013	.041	-0.011	0.014	.444	-0.004	0.014	.762	0.011	0.014	.433	0.015	0.014	.282
Change in Agreeableness	0.002	0.014	.876	0.002	0.014	.906	-0.006	0.014	.655	-0.010	0.014	.484	0.018	0.015	.239	0.014	0.015	.345
Change in Conscientious.	0.033	0.014	.020	0.053	0.014	<.001	0.079	0.014	<.001	0.093	0.014	<.001	0.021	0.015	.151	0.032	0.015	.028
Change in Competencies				0.034	0.014	.017				0.069	0.014	<.001				-0.003	0.018	.887
Change in General Comp.				0.021	0.016	.176				0.022	0.016	.173				-0.002	0.017	.914

Table S5

Standardized Regression Coefficients for the Cross-sectional Models Predicting German, Math, and Science Grades; Separated for Students at Non-Academic and Academic Track Schools

	German						Math						Science					
	Non-Academic Track			Academic Track			Non-Academic Track			Academic Track			Non-Academic Track			Academic Track		
	β	SE	*p*	β	SE	*p*	β	SE	*p*	β	SE	*p*	β	SE	*p*	β	SE	*p*
7th Grade																		
Neuroticism	-0.022	0.021	.301	0.025	0.019	.192	-0.049	0.021	.021	0.015	0.020	.446	-0.041	0.023	.073	0.022	0.023	.347
Extraversion	0.054	0.021	.011	0.081	0.020	<.001	-0.026	0.021	.202	-0.010	0.020	.598	0.027	0.022	.220	-0.005	0.023	.841
Openness	0.018	0.021	.401	0.002	0.020	.927	-0.029	0.020	.153	-0.099	0.020	<.001	-0.009	0.023	.707	-0.046	0.023	.042
Agreeableness	-0.010	0.023	.685	-0.030	0.020	.126	-0.014	0.021	.517	-0.020	0.020	.321	0.042	0.024	.081	-0.016	0.023	.472
Conscientiousness	0.143	0.021	<.001	0.134	0.019	<.001	0.175	0.021	<.001	0.216	0.020	<.001	0.171	0.023	<.001	0.164	0.022	<.001
Competence	0.325	0.022	<.001	0.380	0.020	<.001	0.304	0.024	<.001	0.313	0.021	<.001	0.191	0.032	<.001	0.186	0.027	<.001
General Cog. Comp	-0.013	0.026	.624	0.027	0.022	.222	0.096	0.030	.001	0.151	0.024	<.001	0.051	0.030	.086	0.072	0.025	.003
Migration Status	-0.031	0.021	.129	-0.076	0.019	<.001	0.012	0.021	.580	-0.030	0.020	.140	0.021	0.023	.364	-0.039	0.023	.089
HISEI	0.004	0.025	.883	0.067	0.021	.002	0.024	0.025	.353	0.070	0.022	.002	0.069	0.028	.015	0.074	0.024	.002
Gender	0.118	0.020	.000	0.112	0.020	<.001	-0.021	0.020	.308	0.014	0.021	.496	-0.045	0.023	.044	-0.001	0.022	.974
9th Grade																		
Neuroticism	-0.040	0.021	.065	0.045	0.022	.038	-0.059	0.023	.010	-0.015	0.022	.491	-0.049	0.024	.037	-0.022	0.023	.329
Extraversion	0.057	0.021	.007	0.075	0.021	<.001	-0.051	0.021	.016	-0.026	0.021	.218	0.010	0.023	.658	-0.014	0.021	.492
Openness	0.034	0.020	.093	0.052	0.021	.013	-0.025	0.021	.248	-0.085	0.020	<.001	-0.020	0.024	.389	-0.057	0.022	.008
Agreeableness	0.021	0.021	.317	-0.035	0.021	.104	-0.006	0.022	.790	0.000	0.020	.996	0.018	0.024	.453	-0.029	0.022	.177
Conscientiousness	0.178	0.020	<.001	0.160	0.021	<.001	0.168	0.021	<.001	0.213	0.020	<.001	0.189	0.022	<.001	0.224	0.021	<.001
Competence	0.263	0.023	<.001	0.313	0.022	<.001	0.283	0.023	<.001	0.398	0.020	<.001	0.190	0.024	<.001	0.270	0.022	<.001
General Cog. Comp.	-0.010	0.023	.667	0.003	0.021	.902	0.071	0.024	.004	0.078	0.021	<.001	0.066	0.024	.007	0.100	0.022	<.001
Migration Status	-0.014	0.020	.498	-0.099	0.020	<.001	0.023	0.022	.287	0.027	0.019	.162	0.007	0.023	.773	-0.020	0.021	.338
HISEI	0.085	0.025	.001	0.098	0.022	<.001	0.045	0.027	.095	0.066	0.022	.003	0.069	0.030	.020	0.043	0.023	.061
Gender	0.179	0.020	<.001	0.165	0.021	<.001	0.038	0.021	.074	0.045	0.021	.033	0.011	0.023	.639	-0.031	0.022	.164

Table S6

Class-centered, Standardized Regression Coefficients for the Cross-sectional Models Predicting German, Math, and Science Grades

	7th Grade									9th Grade								
	Model $1_{centered}$			Model $2_{centered}$			Model $3_{centered}$			Model $1_{centered}$			Model $2_{centered}$			Model $3_{centered}$		
	β	SE	p	β	SE	p	β	SE	p	β	SE	p	β	SE	p	β	SE	p
German																		
Neuroticism	0.014	0.014	.339	0.015	0.014	.287	-0.008	0.014	.575	0.044	0.017	.009	0.039	0.016	.016	-0.003	0.017	.883
Extraversion	0.062	0.015	<.001	0.047	0.014	.001	0.042	0.014	.003	0.040	0.017	.017	0.049	0.016	.003	0.042	0.016	.009
Openness	0.059	0.014	<.001	0.024	0.014	.094	0.006	0.014	.681	0.081	0.016	<.001	0.057	0.016	<.001	0.030	0.016	.061
Agreeableness	0.002	0.015	.906	-0.001	0.015	.953	-0.013	0.015	.365	0.000	0.018	.998	0.002	0.017	.893	-0.011	0.017	.495
Conscientiousness	0.199	0.015	<.001	0.172	0.014	<.001	0.159	0.014	<.001	0.227	0.016	<.001	0.222	0.016	<.001	0.203	0.016	<.001
German Comp.				0.311	0.015	<.001	0.296	0.016	<.001				0.299	0.018	<.001	0.259	0.020	<.001
General Cog. Comp				-0.072	0.018	<.001	-0.058	0.019	.002				-0.077	0.019	<.001	-0.069	0.019	<.001
Migration Status							-0.043	0.015	.004							-0.046	0.017	.008
HISEI							-0.036	0.017	.041							0.011	0.020	.570
Gender							0.119	0.014	<.001							0.164	0.017	<.001
R^2		.050			.129			.144			.065			.139			.164	
Math																		
Neuroticism	-0.049	0.015	.001	-0.018	0.014	.214	-0.011	0.015	.432	-0.064	0.018	<.001	-0.033	0.017	.050	-0.034	0.017	.052
Extraversion	-0.047	0.015	.001	-0.043	0.014	.002	-0.039	0.014	.006	-0.083	0.017	<.001	-0.058	0.017	<.001	-0.056	0.017	.001
Openness	-0.034	0.014	.017	-0.067	0.014	<.001	-0.059	0.014	<.001	-0.035	0.017	.037	-0.051	0.016	.001	-0.051	0.016	.002
Agreeableness	-0.025	0.015	.101	-0.014	0.014	.340	-0.009	0.015	.546	-0.028	0.018	.106	-0.012	0.017	.452	-0.012	0.017	.456
Conscientiousness	0.181	0.015	<.001	0.188	0.014	<.001	0.194	0.014	<.001	0.192	0.017	<.001	0.208	0.016	<.001	0.207	0.016	<.001
Math Comp.				0.247	0.017	<.001	0.246	0.019	<.001				0.285	0.018	<.001	0.303	0.020	<.001
General Cog. Comp				0.092	0.020	<.001	0.094	0.020	<.001				0.038	0.020	.056	0.041	0.020	.038
Migration Status							-0.008	0.016	.608							0.026	0.018	.141
HISEI							-0.022	0.018	.215							-0.039	0.021	.064
Gender							-0.039	0.014	.005							0.006	0.017	.725
R^2		.035			.126			.128			.042			.134			.136	
Science																		
Neuroticism	-0.039	0.017	.018	-0.018	0.017	.291	-0.010	0.017	.552	-0.077	0.017	<.001	-0.052	0.017	.003	-0.036	0.018	.038
Extraversion	-0.014	0.017	.403	-0.008	0.017	.644	-0.007	0.017	.686	-0.032	0.017	.065	-0.013	0.017	.455	-0.010	0.017	.552
Openness	-0.002	0.016	.911	-0.029	0.016	.074	-0.019	0.017	.265	-0.025	0.018	.158	-0.060	0.017	.001	-0.049	0.018	.005
Agreeableness	-0.010	0.017	.576	0.005	0.017	.782	0.009	0.017	.623	-0.039	0.018	.031	-0.025	0.018	.156	-0.021	0.018	.251
Conscientiousness	0.159	0.016	<.001	0.168	0.016	<.001	0.174	0.016	<.001	0.195	0.017	<.001	0.218	0.016	<.001	0.225	0.017	<.001
Science Comp.				0.177	0.022	<.001	0.158	0.024	<.001				0.213	0.019	<.001	0.209	0.020	<.001
General Cog. Comp				0.020	0.022	.370	0.018	0.022	.416				0.049	0.020	.013	0.052	0.020	.009
Migration Status							-0.037	0.018	.039							-0.011	0.019	.550
HISEI							0.015	0.020	.465							-0.006	0.021	.765
Gender							-0.050	0.016	.002							-0.057	0.017	.001
R^2		.027			.060			.064			.042			.096			0.099	

Figure S1

Standardized Estimates for the Cross-Sectional Models, Separated for Students at Non-Academic ad Academic Track Schools

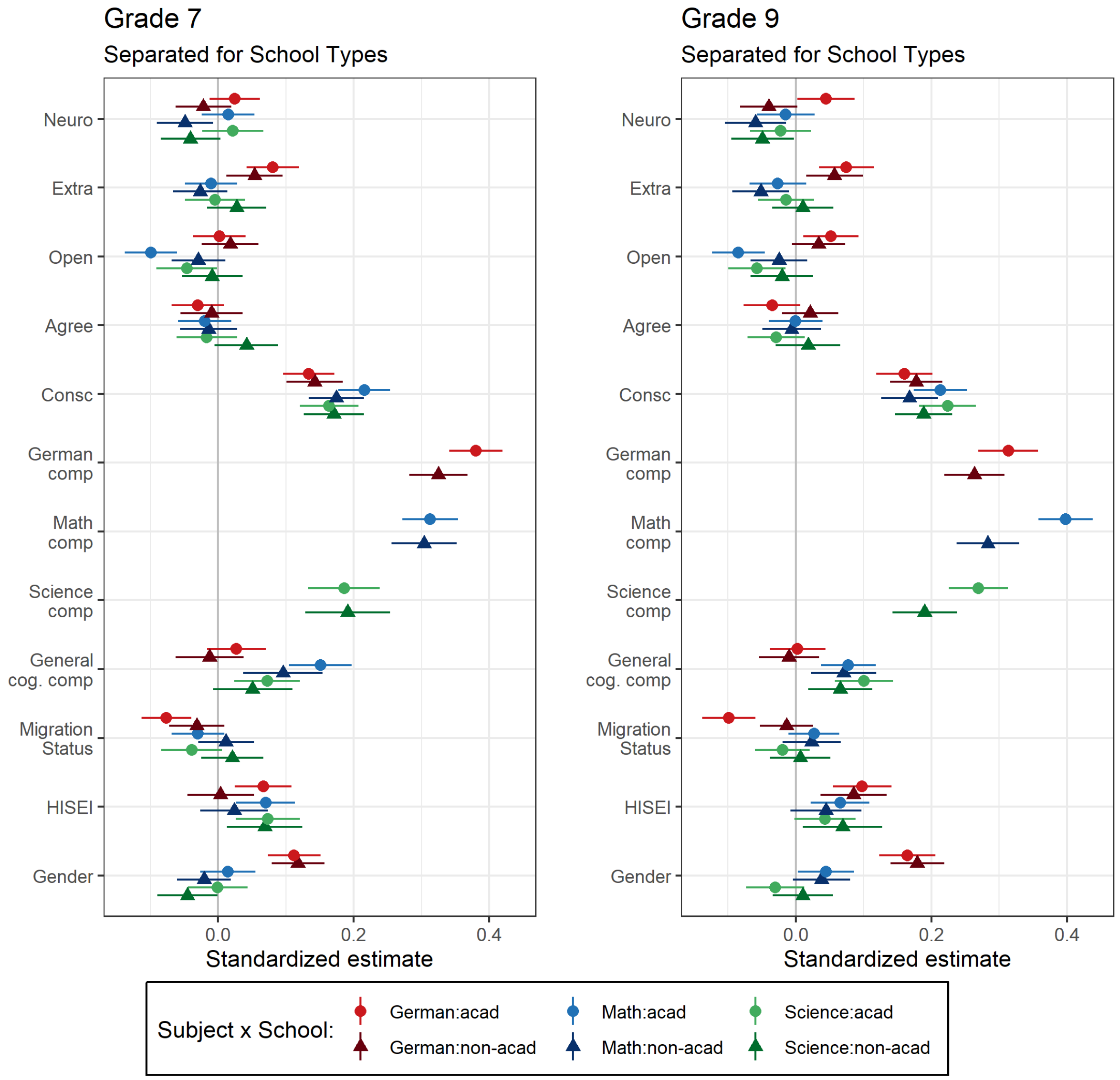

Note. Results are shown for the most comprehensive model M3, separately for the subjects and school types.

Neuro = neuroticism; Extra = extraversion; Open = openness; Agree = agreeableness; Consc = conscientiousness; comp = competence; cog = cognitive; HISEI = Highest International Socio-Economic Index of Occupational Status.

Figure S2

Standardized Estimates for the Cross-Sectional Models, Grades Centered Within Class

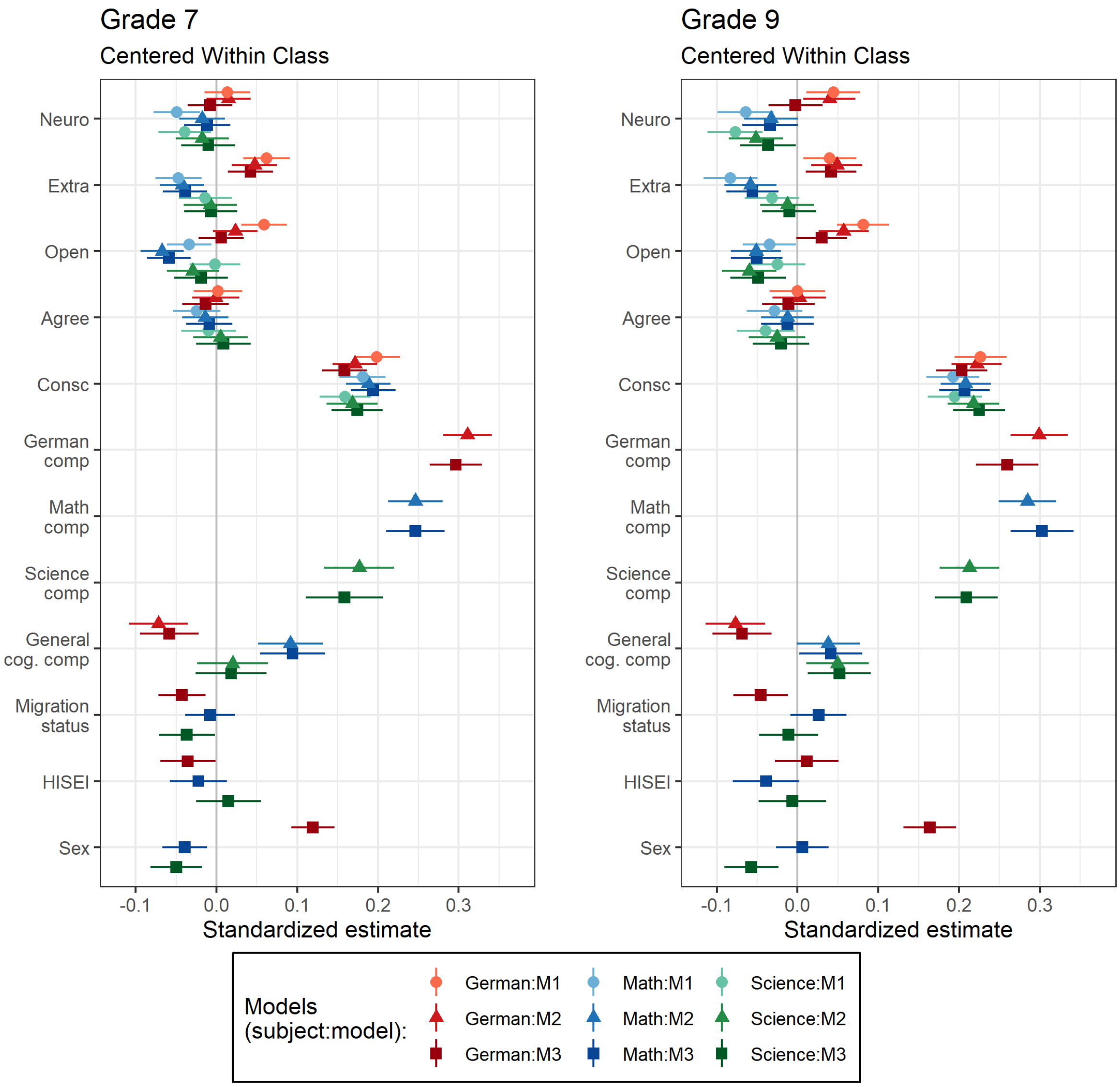

Note. Results are shown for the increasingly comprehensive models M1–M3, separately for the subjects and grade levels.

Neuro = neuroticism; Extra = extraversion; Open = openness; Agree = agreeableness; Consc = conscientiousness; comp = competence; cog = cognitive, HISEI = Highest International Socio-Economic Index of Occupational Status.
